# Real-world comparative effectiveness of FOLFOX, FOLFIRI, and CapOx as second-line chemotherapy for advanced biliary tract cancer

**DOI:** 10.1093/oncolo/oyag424

**Published:** 2026-10-21

**Authors:** Chun-Yang Chao, Kuan-Yu Tseng, Hsin-Chen Lin, Yu-Hsuan Shih

**Affiliations:** Division of Medical Oncology, Department of Oncology, Taichung Veterans General Hospital, Taichung 407219, Taiwan; Division of Medical Oncology, Department of Oncology, Taichung Veterans General Hospital, Taichung 407219, Taiwan; Division of Medical Oncology, Department of Oncology, Taichung Veterans General Hospital, Taichung 407219, Taiwan; Department of Post-Baccalaureate Medicine, College of Medicine, National Chung Hsing University, Taichung 402202, Taiwan; Division of Medical Oncology, Department of Oncology, Taichung Veterans General Hospital, Taichung 407219, Taiwan; Department of Post-Baccalaureate Medicine, College of Medicine, National Chung Hsing University, Taichung 402202, Taiwan; Institute of Medicine, College of Medicine, Chung Shan Medical University, Taichung 402306, Taiwan

**Keywords:** biliary tract cancer, second-line chemotherapy, real-world evidence, FOLFOX, FOLFIRI, CapOx

## Abstract

**Background:**

Advanced biliary tract cancer (BTC) has a poor prognosis following progression on first-line gemcitabine plus cisplatin. However, evidence comparing second-line chemotherapy regimens remains limited. This study aimed to compare the effectiveness and safety of fluorouracil, leucovorin, and oxaliplatin (FOLFOX); fluorouracil, leucovorin, and irinotecan (FOLFIRI); and capecitabine plus oxaliplatin (CapOx).

**Patients and Methods:**

Adults with advanced BTC who received first-line gemcitabine plus cisplatin followed by second-line FOLFOX, FOLFIRI, or CapOx between January 2010 and July 2025 were identified from the TriNetX Global Collaborative Network. Patients who subsequently received molecularly targeted therapies were excluded. The study included 825 patients receiving FOLFOX, 184 receiving FOLFIRI, and 149 receiving CapOx; after 1:1 propensity score matching, the respective comparisons included 182 and 149 patients per group. Overall survival (OS) and treatment-related adverse events were compared between the matched cohorts.

**Results:**

The median OS was comparable between FOLFOX and FOLFIRI (5.7 months vs 5.8 months, *P* = .958) and between FOLFOX and CapOx (5.7 months vs 5.8 months, *P* = .803). The overall adverse event profiles were also comparable. However, FOLFOX was associated with lower rates of neutropenia but higher rates of thrombocytopenia than FOLFIRI. In contrast, FOLFOX and CapOx demonstrated similar toxicity despite modest differences in anemia and thrombocytopenia.

**Conclusion:**

This real-world study demonstrates comparable survival outcomes among FOLFOX, FOLFIRI, and CapOx as second-line chemotherapies for advanced BTC. These findings support individualized regimen selection based on patient characteristics and toxicity profiles.

Implications for practice There is limited comparative evidence to guide second-line chemotherapy selection after progression on gemcitabine plus cisplatin in advanced biliary tract cancer. This real-world propensity score-matched study found no clinically meaningful differences in survival among FOLFOX, FOLFIRI, and CapOx, while differences in toxicity were modest. These findings support individualized regimen selection based on patient characteristics and toxicity profiles in clinical practice.

## Introduction

Biliary tract cancers (BTCs), including intrahepatic cholangiocarcinoma (iCCA), extrahepatic cholangiocarcinoma (eCCA), and gallbladder cancer (GBC), are relatively rare but highly aggressive malignancies with an increasing global burden. A total of 216 768 incident cases and 171 961 related deaths were reported worldwide in 2021, accounting for a 101% increase in incidence and a 74% increase in mortality from 1990.^1^^,^^2^ The incidence of BTC varies across geographic regions, with the highest rates observed in Asia. Given that early-stage disease is often asymptomatic or associated with nonspecific symptoms, most patients are diagnosed at an advanced stage, with approximately 70%-80% presenting with locally advanced or metastatic disease at the time of diagnosis.^3^ Importantly, advanced BTC has a poor prognosis. A population-based study using the Surveillance, Epidemiology, and End Results database reported a median overall survival (OS) of less than 1 year for patients with advanced disease.^4^

Systemic therapy remains the cornerstone of treatment for advanced BTC, with gemcitabine plus cisplatin established as the historical standard first-line treatment based on the results of the ABC-02 trial.^5^ More recently, 2 pivotal phase III trials, TOPAZ-1 and KEYNOTE-966, have demonstrated an OS benefit with the addition of durvalumab and pembrolizumab, respectively, to chemotherapy, establishing chemoimmunotherapy as a contemporary first-line standard of care.^6^^,^^7^ Recent real-world studies have further supported the effectiveness of chemoimmunotherapy in the first-line treatment of advanced BTC.^8^^,^^9^ As the use of immune checkpoint inhibitors expands, increasing attention has also been directed toward patient-related factors and treatment-associated toxicities that may influence their clinical use.^10–12^ However, the prognosis remains poor following disease progression after first-line treatment. Contemporary real-world evidence suggests that approximately one-third of patients initiating first-line systemic therapy proceed to second-line treatment, although median OS among patients receiving subsequent therapy remains approximately 5-7 months.^13–15^

Current international guidelines recommend comprehensive molecular profiling to identify actionable genomic alterations for targeted therapy. Nevertheless, actionable alterations are identified in only approximately 50% of patients.^16^^,^^17^ Moreover, the application of molecularly targeted therapy is often limited by restricted access to molecular testing, drug availability, and the financial burden associated with genomic testing and targeted agent treatment.^18^ Therefore, chemotherapy remains the predominant second-line treatment strategy for most patients with advanced BTC.

However, chemotherapeutic options beyond the first-line therapy are still limited. Fluorouracil, leucovorin, and oxaliplatin (FOLFOX) is established as a standard second-line regimen based on the ABC-06 trial demonstrating its superior OS benefit in comparison to active symptom control alone.^19^ Irinotecan-based chemotherapy regimens are also recommended by current treatment guidelines based on evidence from phase II prospective studies and retrospective analyses showing modest clinical activity of irinotecan-containing treatments for advanced BTC.^20^^,^^21^ Nevertheless, no phase III trial has directly compared FOLFOX with irinotecan-based chemotherapy in the second-line setting.

Capecitabine plus oxaliplatin (CapOx) has been widely used to treat gastrointestinal malignancies, such as gastric and colorectal cancers.^22^^,^^23^ However, data supporting its use in advanced BTC are limited. There remains a major evidence gap regarding the comparative efficacy of FOLFOX and other doublet chemotherapy regimens in the subsequent-line treatment of BTC. This study aimed to evaluate the effectiveness and tolerability of fluorouracil, leucovorin, and irinotecan (FOLFIRI) and CapOx as alternative second-line treatment options. Toward this goal, data from the TriNetX Global Collaborative Network were analyzed to compare FOLFIRI and CapOx with FOLFOX in the second-line setting in advanced BTC.

## Methods

### Study design and data source

This retrospective observational study was approved by the Institutional Review Board of Taichung Veterans General Hospital (IRB No. CE26323C) and was conducted in accordance with the Declaration of Helsinki.

Data from the TriNetX Global Collaborative Network were analyzed. Briefly, the TriNetX Global Collaborative Network provides access to electronic medical records (diagnoses, procedures, medications, and laboratory values) from approximately 179 537 628 patients across 172 healthcare organizations. The platform contains de-identified patient-level information derived from routine clinical practice, including demographic characteristics, diagnoses, procedures, medication records, laboratory results, and survival data. Access to the database is restricted to aggregated, de-identified datasets and complies with the Health Insurance Portability and Accountability Act.

### Data collection and study population

The data used in this study were collected on February 17, 2026. Adult patients (≥18 years) diagnosed with BTC, particularly iCCA, eCCA, and GBC, between January 1, 2010 and July 31, 2025 were identified using the International Classification of Diseases, 10th Revision, Clinical Modification (ICD-10-CM) codes C22.1, C24.0, and C23, respectively. Patients were required to have received second-line treatment with FOLFOX, FOLFIRI, or CapOx after failure of first-line gemcitabine plus cisplatin. Concomitant use of immune checkpoint inhibitors with first-line gemcitabine plus cisplatin did not preclude study inclusion. To exclusively evaluate the comparative effectiveness of conventional cytotoxic chemotherapy, patients who subsequently received molecularly targeted therapies were excluded. The study period commenced in 2010 following the establishment of gemcitabine plus cisplatin as the standard first-line regimen for advanced BTC. The 2025 end date was selected to ensure sufficient follow-up before data extraction.

As tumor-node-metastasis (TNM) staging information was not uniformly available within the TriNetX platform, advanced BTC was defined as the presence of lymph node involvement or distant organ metastases identified using relevant ICD diagnostic codes. Treatment exposure was determined using RxNorm medication records and Healthcare Common Procedure Coding System procedure codes. The patients were subsequently classified according to the second-line chemotherapy regimen received: FOLFOX, FOLFIRI, or CapOx. Detailed coding algorithms for the diagnoses, treatments, and outcomes are provided in Table S1.

### Treatment and outcomes

Treatment initiation was designated as the index date and corresponded to the earliest date on which all agents comprising the selected regimen had been administered. Comparative effectiveness analyses were performed separately for the FOLFOX vs FOLFIRI and FOLFOX vs CapOx groups.

The primary outcome measure was OS, which was measured from the index date to death from any cause. Patients without a recorded death were censored on the day after their last recorded clinical fact within the analysis window, according to the TriNetX survival analysis methodology. The secondary outcome measures were treatment-related adverse events, including hematologic toxicities (eg, anemia, neutropenia, and thrombocytopenia), sepsis, acute kidney injury, peripheral neuropathy, constipation, diarrhea, malaise and fatigue, nausea or vomiting, dermatitis or rash, hyponatremia, and hypokalemia. Adverse events were identified using ICD-10-CM diagnostic codes, Logical Observation Identifier Names and Codes laboratory codes, and predefined laboratory thresholds available within the TriNetX platform. Grade 3 hematologic toxicities were defined using laboratory thresholds consistent with the National Cancer Institute Common Terminology Criteria for Adverse Events (CTCAE), version 5.0, whereas grade 3 electrolyte abnormalities were identified using prespecified laboratory thresholds in the TriNetX platform. Detailed definitions and thresholds for individual adverse events are provided in Table S1.

### Statistical analysis

Continuous variables are summarized as the mean ± standard deviation (SD) and categorical variables as frequencies and percentages. Baseline characteristics were compared between the treatment groups using Student’s *t* test for continuous variables and the chi-square test for categorical variables, as appropriate. To reduce the impact of potential confounding, separate 1:1 propensity score matching analyses were performed for the FOLFOX vs FOLFIRI and FOLFOX vs CapOx cohorts. Demographic characteristics, comorbidities, metastatic sites (lymph nodes, lung, and peritoneum/retroperitoneum), and tumor marker levels were included in the matching algorithm. Propensity score matching was performed using 1:1 greedy nearest-neighbor matching with a caliper of 0.1 pooled standard deviations. Laboratory covariates defined by categorical thresholds were represented as binary indicators. Patients meeting a specified threshold were coded as present, whereas those not meeting the threshold, including patients without a recorded value, were coded as absent. Therefore, patients with missing laboratory data were not excluded from propensity score matching, and no numerical imputation of missing laboratory values was performed. Baseline covariate balance was evaluated using standardized mean differences. Propensity score distributions before and after matching for the FOLFOX vs FOLFIRI and FOLFOX vs CapOx comparisons are shown in Figures S1 and S2 (see online supplementary material for a color version of these figures), respectively. OS was estimated using the Kaplan–Meier method and compared between treatment groups using stratified log-rank tests. Hazard ratios (HRs) and the corresponding 95% CIs were calculated using stratified Cox proportional hazards regression models. All statistical analyses were conducted using the TriNetX web-based analytics platform (TriNetX LLC). All tests were 2-sided, and *P* < .05 was considered statistically significant.

## Results

### Selected patients

A total of 144 768 patients with BTC were identified. Among 44 350 adult patients with advanced disease, 5134 had received first-line gemcitabine plus cisplatin. Following exclusion of patients who subsequently received molecularly targeted therapies, 1158 patients who received FOLFOX (*n* = 825), FOLFIRI (*n* = 184), or CapOx (*n* = 149) as second-line chemotherapy were included for propensity score matching. Finally, 182 patients per group were included in the FOLFOX vs FOLFIRI comparison, while 149 patients per group were included in the FOLFOX vs CapOx comparison. The patient selection process is illustrated in Figure 1. After propensity score matching, the median follow-up durations were 146 and 176 days for the FOLFOX and FOLFIRI cohorts, respectively, and 161 and 162 days for the FOLFOX and CapOx cohorts, respectively.

**Figure 1.**
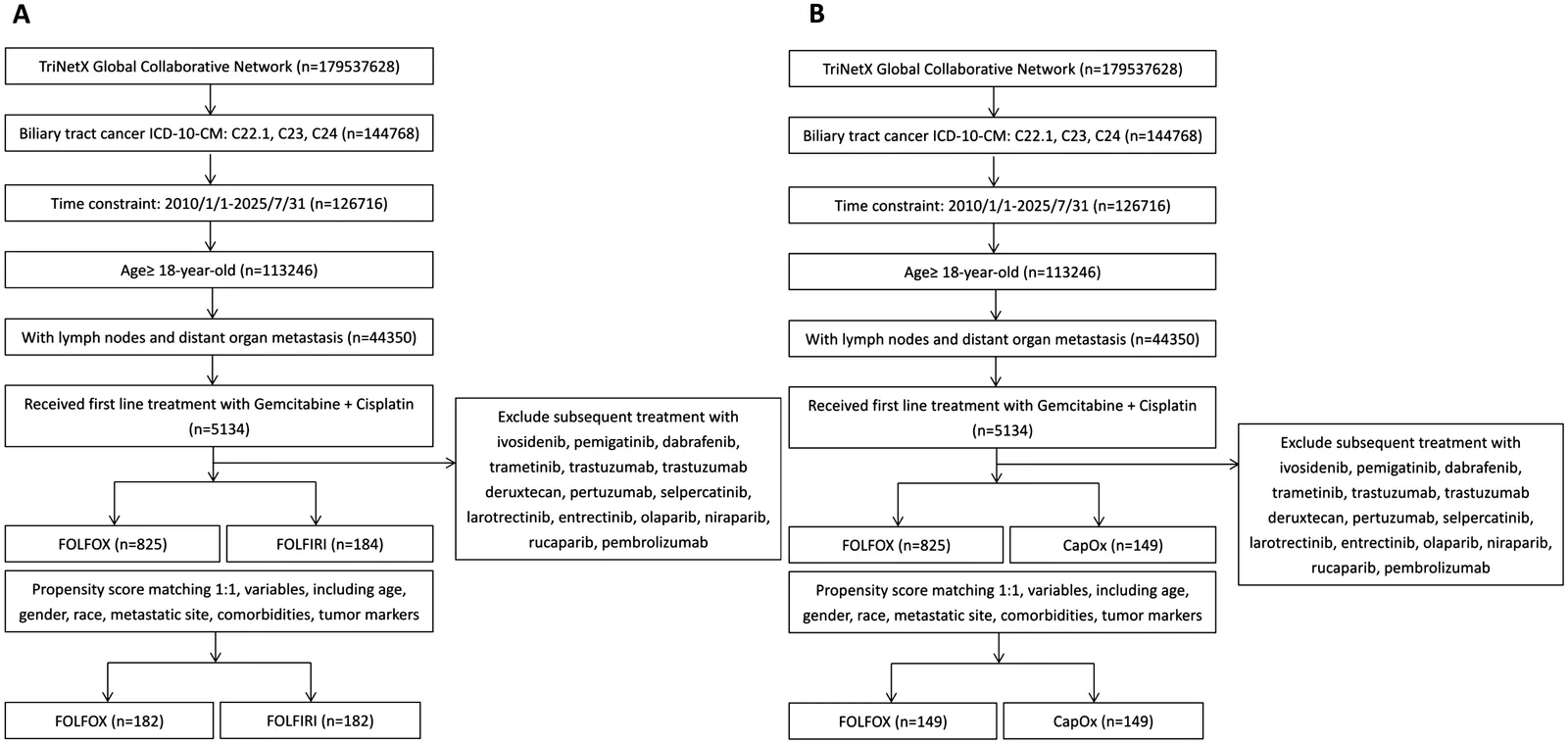
Patient selection flowcharts. (A) Patient selection process for the FOLFOX vs FOLFIRI cohort. (B) Patient selection process for the FOLFOX vs CapOx cohort. Both flowcharts illustrate patient identification, eligibility criteria, exclusion of patients who subsequently received molecularly targeted therapies, and 1:1 propensity score matching.

### FOLFOX vs FOLFIRI

#### Baseline characteristics

The baseline characteristics of the FOLFOX and FOLFIRI groups are summarized in Table 1. Most baseline characteristics were comparable between the 2 groups, with no significant differences in age, sex, major comorbidities, other metastatic sites, or baseline tumor marker levels. However, the FOLFOX group more frequently had lymph node metastases (27.8% vs 20.1%, *P* = .033), whereas the FOLFIRI group less frequently had patients of other races (2.2% vs 6.5%, *P* = .021). Following 1:1 propensity score matching, 182 patients were included in each cohort, and baseline characteristics were well balanced. Tumor marker availability before and after matching is reported in Table 1.

**Table 1.** Baseline characteristics of the patients in the FOLFOX and FOLFIRI groups, overall cohort and propensity-matched cohort.

	Overall cohort				Propensity-matched cohort			
	FOLFOX (*n* = 825)	FOLFIRI (*n* = 184)	*P-*value	SMD	FOLFOX (*n* = 182)	FOLFIRI (*n* = 182)	*P-*value	SMD
**Age at index, mean ± SD**	63.6 ± 10.2	62.5 ± 11.1	.217	0.098	63.3 ± 9.7	62.6 ± 11.2	.510	0.069
** <65, *n* (%)**	403 (48.8)	101 (54.9)	.138	0.121	98 (53.8)	99 (54.4)	.916	0.011
** ≥65, *n* (%)**	422 (51.2)	83 (45.1)	.138	0.121	84 (46.2)	83 (45.6)	.916	0.011
**Sex, *n* (%)**								
** Female**	425 (51.5)	98 (53.3)	.668	0.035	93 (51.1)	97 (53.3)	.675	0.044
** Male**	400 (48.5)	86 (46.7)	.668	0.035	89 (48.9)	85 (46.7)	.675	0.044
**Race, *n* (%)**								
** White**	515 (62.4)	123 (66.8)	.260	0.093	107 (58.8)	121 (66.8)	.129	0.160
** Black or African American**	70 (8.5)	15 (8.2)	.883	0.012	24 (13.2)	15 (8.2)	.127	0.160
** Asian**	87 (10.5)	21 (11.4)	.731	0.028	23 (12.6)	21 (11.5)	.748	0.034
** Unknown race**	99 (12)	21 (11.4)	.824	0.018	25 (13.7)	21 (11.5)	.528	0.066
** Other race**	54 (6.5)	4 (2.2)	.021	0.188	3 (1.6)	4 (2.2)	.703	0.040
**Comorbidities, *n* (%)**								
** Diabetes mellitus**	186 (22.5)	43 (23.4)	.809	0.020	51 (28.0)	42 (23.1)	.279	0.114
** Ischemic heart disease**	126 (15.3)	22 (12.0)	.250	0.097	23 (12.6)	22 (12.1)	.873	0.017
** Chronic lower respiratory disease**	114 (13.8)	21 (11.4)	.386	0.072	21 (11.5)	21 (11.5)	1	<0.001
** Chronic kidney disease**	81 (9.8)	12 (6.5)	.162	0.121	13 (7.1)	12 (6.6)	.836	0.022
**Site of metastasis, *n* (%)**								
** Lymph nodes**	229 (27.8)	37 (20.1)	.033	0.180	36 (19.8)	37 (20.3)	.896	0.014
** Lung**	133 (16.1)	26 (14.1)	.503	0.056	27 (14.8)	26 (14.3)	.882	0.016
** Peritoneum or retroperitoneum**	170 (20.6)	36 (19.6)	.751	0.026	39 (21.4)	36 (19.8)	.697	0.041
**CEA level, *n* (%)**								
** Available**	366 (44.4)	93 (50.5)			87 (47.8)	90 (49.5)		
** Normal**	207 (25.1)	50 (27.2)	.558	0.047	50 (27.5)	49 (26.9)	.906	0.012
** Elevated (≥5 U/mL)**	159 (19.3)	43 (23.4)	.209	0.100	37 (20.3)	41 (22.5)	.609	0.054
**CA 19-9 level, *n* (%)**								
** Available**	581 (70.4)	118 (64.1)			112 (61.5)	117 (64.3)		
** Normal**	177 (21.5)	32 (17.4)	.219	0.103	32 (17.6)	32 (17.6)	1	<0.001
** Elevated (≥37 U/mL)**	404 (49.0)	86 (46.7)	.584	0.045	80 (44.0)	85 (46.7)	.599	0.055

Abbreviations: CA 19-9 = carbohydrate antigen 19-9; CEA = carcinoembryonic antigen; FOLFIRI = fluorouracil, leucovorin, and irinotecan; FOLFOX = fluorouracil, leucovorin, and oxaliplatin; SMD = standardized mean differences.

#### Survival analysis and adverse events

The median OS was nearly identical between the FOLFOX and FOLFIRI groups following propensity score matching (5.7 months vs 5.8 months; log-rank *P* = .958; Figure 2). In addition, adverse event profiles were generally comparable between the 2 groups (Figure 3). There were no significant differences in the incidence rates of anemia, sepsis, acute kidney injury, peripheral neuropathy, gastrointestinal toxicities, dermatitis or rash, grade 3 hyponatremia, or grade 3 hypokalemia between the groups. However, thrombocytopenia occurred more frequently in the FOLFOX group than in the FOLFIRI group (77.5% vs 72.0%; HR: 1.43, 95% CI: 1.13-1.82), whereas neutropenia (56.6% vs 72.5%; HR: 0.67, 95% CI: 0.52-0.87) and grade 3 neutropenia (24.2% vs 36.3%; HR: 0.65, 95% CI: 0.44-0.95) were more common in the FOLFIRI group.

**Figure 2.**
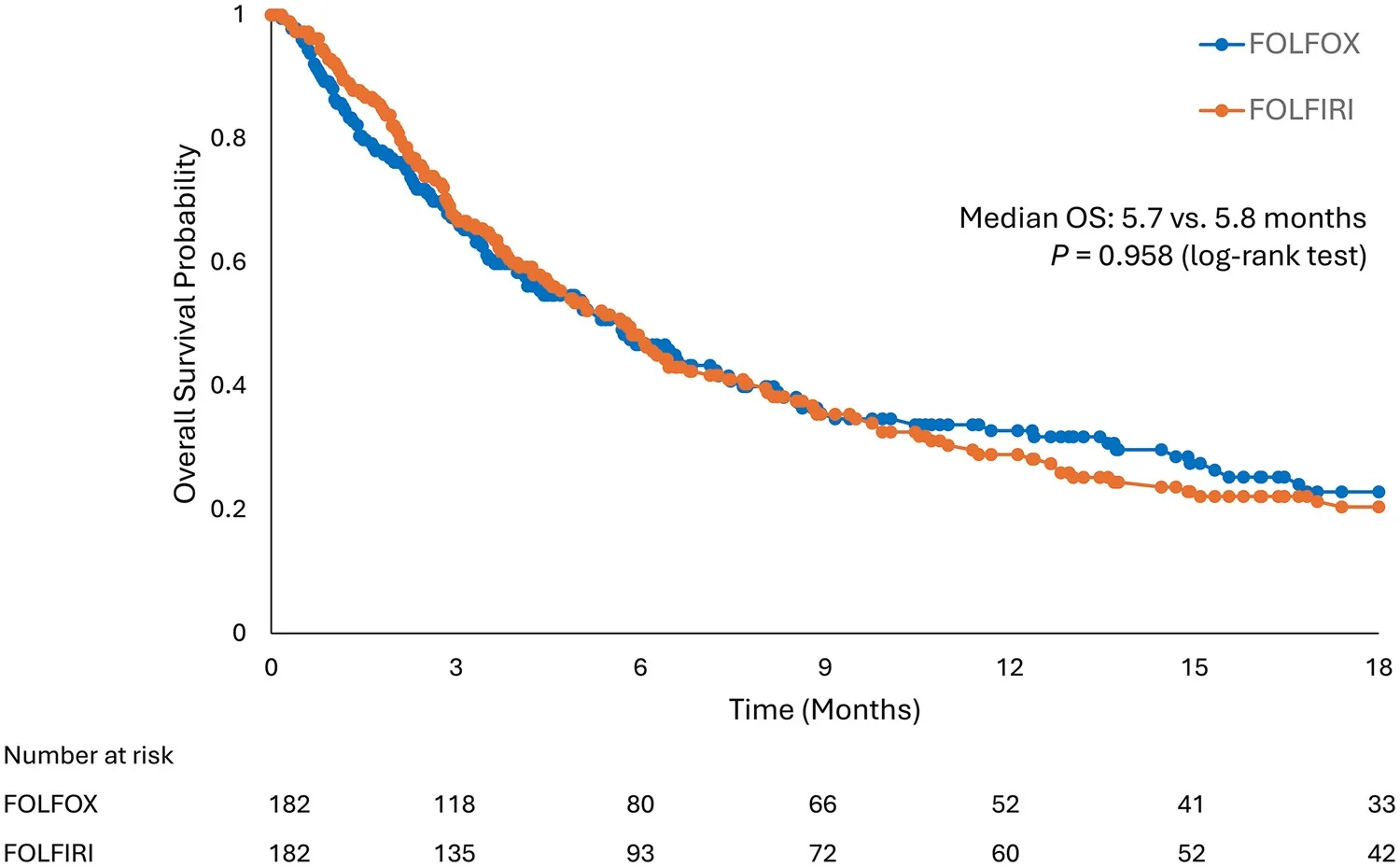
Overall survival of the propensity score-matched FOLFOX and FOLFIRI cohorts. Kaplan-Meier curves comparing overall survival between patients treated with FOLFOX and FOLFIRI after propensity score matching.

**Figure 3.**
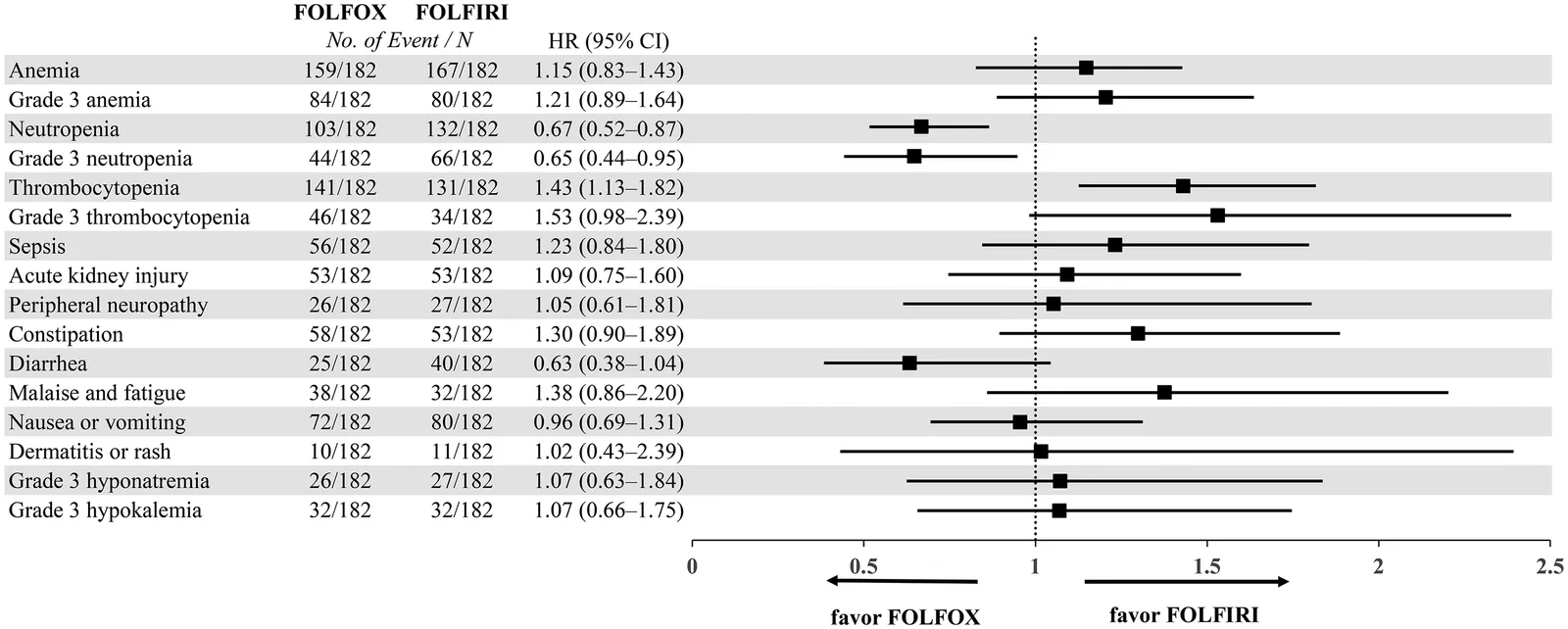
Adverse events in the propensity score-matched FOLFOX and FOLFIRI cohorts. Forest plot comparing treatment-related adverse events between the FOLFOX and FOLFIRI groups after propensity score matching.

### FOLFOX vs CapOx

#### Baseline characteristics

The baseline characteristics of the FOLFOX and CapOx cohorts are summarized in Table 2. Most baseline characteristics were comparable between the 2 treatment groups before propensity score matching, with no significant differences in age, sex, race, major comorbidities, metastatic sites, or carbohydrate antigen 19-9 levels. However, the proportion of patients with normal carcinoembryonic antigen levels was lower in the CapOx group (16.1% vs 25.1%, *P* = .018). Following 1:1 propensity score matching, 149 patients were included in each group, and the baseline characteristics remained well balanced, with no clinically meaningful differences observed. Tumor marker availability before and after matching is reported in Table 2.

**Table 2.** Baseline characteristics of the patients in the FOLFOX and CapOx groups, overall cohort and propensity-matched cohort.

	Overall cohort				Propensity-matched cohort			
	FOLFOX (*n* = 825)	CapOx (*n* = 149)	*P-*value	SMD	FOLFOX (*n* = 149)	CapOx (*n* = 149)	*P-*value	SMD
**Age at index, mean ± SD**	63.6 ± 10.2	64.0 ± 10.5	.670	0.038	63.3 ± 10.8	64.0 ± 10.5	.617	0.058
** <65, *n* (%)**	403 (48.8)	68 (45.6)	.470	0.064	73 (49.0)	68 (45.6)	.562	0.067
** ≥65, *n* (%)**	422 (51.2)	81 (54.4)	.470	0.064	76 (51.0)	81 (54.4)	.562	0.067
**Sex, *n* (%)**								
** Female**	425 (51.5)	85 (57.0)	.213	0.111	80 (53.7)	85 (57.0)	.560	0.068
** Male**	400 (48.5)	64 (43.0)	.213	0.111	69 (46.3)	64 (43.0)	.560	0.069
**Race, *n* (%)**								
** White**	515 (62.4)	88 (59.1)	.436	0.069	89 (59.7)	88 (59.1)	.906	0.014
** Black or African American**	70 (8.5)	13 (8.7)	.923	0.009	13 (8.7)	13 (8.7)	1	<0.001
** Asian**	87 (10.5)	17 (11.4)	.753	0.028	16 (10.7)	17 (11.4)	.854	0.021
** Unknown race**	99 (12)	22 (14.8)	.346	0.081	25 (16.8)	22 (14.8)	.633	0.055
** Other race**	54 (6.5)	9 (6.0)	.818	0.021	6 (4.0)	9 (6.0)	.427	0.092
**Comorbidities, *n* (%)**								
** Diabetes mellitus**	186 (22.5)	29 (19.5)	.404	0.076	31 (20.8)	29 (19.5)	.773	0.033
** Ischemic heart disease**	126 (15.3)	14 (9.4)	.060	0.179	15 (10.1)	14 (9.4)	.845	0.023
** Chronic lower respiratory disease**	114 (13.8)	15 (10.1)	.214	0.116	19 (12.8)	15 (10.1)	.466	0.085
** Chronic kidney disease**	81 (9.8)	10 (6.7)	.230	0.113	10 (6.7)	10 (6.7)	1	<0.001
**Site of metastasis, *n* (%)**								
** Lymph nodes**	229 (27.8)	36 (24.2)	.364	0.082	31 (20.8)	36 (24.2)	.488	0.080
** Lung**	133 (16.1)	16 (10.7)	.230	0.113	13 (8.7)	16 (10.7)	.558	0.068
** Peritoneum or retroperitoneum**	170 (20.6)	22 (14.8)	.099	0.154	23 (15.4)	22 (14.8)	.871	0.019
**CEA level, *n* (%)**								
** Available**	366 (44.4)	43 (28.9)			42 (28.2)	43 (28.9)		
** Normal**	207 (25.1)	24 (16.1)	.018	0.224	25 (16.8)	24 (16.1)	.876	0.018
** Elevated (≥5 U/mL)**	159 (19.3)	19 (12.8)	.058	0.179	17 (11.4)	19 (12.8)	.722	0.041
**CA 19-9 level, *n* (%)**								
** Available**	581 (70.4)	110 (73.8)			113 (75.8)	110 (73.8)		
** Normal**	177 (21.5)	35 (23.5)	.579	0.049	33 (22.1)	35 (23.5)	.782	0.032
** Elevated (≥37 U/mL)**	404 (49.0)	75 (50.3)	.759	0.027	80 (53.7)	75 (50.3)	.562	0.067

Abbreviations: CA 19-9 = carbohydrate antigen 19-9; CapOx = capecitabine and oxaliplatin; CEA = carcinoembryonic antigen; FOLFOX = fluorouracil, leucovorin, and oxaliplatin; SMD = standardized mean differences.

#### Survival analysis and adverse events

The median OS after propensity score matching was nearly identical between the FOLFOX and CapOx groups (5.7 months vs 5.8 months; log-rank *P* = .803; Figure 4). In addition, overall adverse event profiles were comparable between the 2 groups (Figure 5). Although anemia and thrombocytopenia were observed more frequently in the FOLFOX group, the incidence rates of grade 3 hematologic toxicities were comparable. There were also no major differences in the incidence of non-hematologic adverse events.

**Figure 4.**
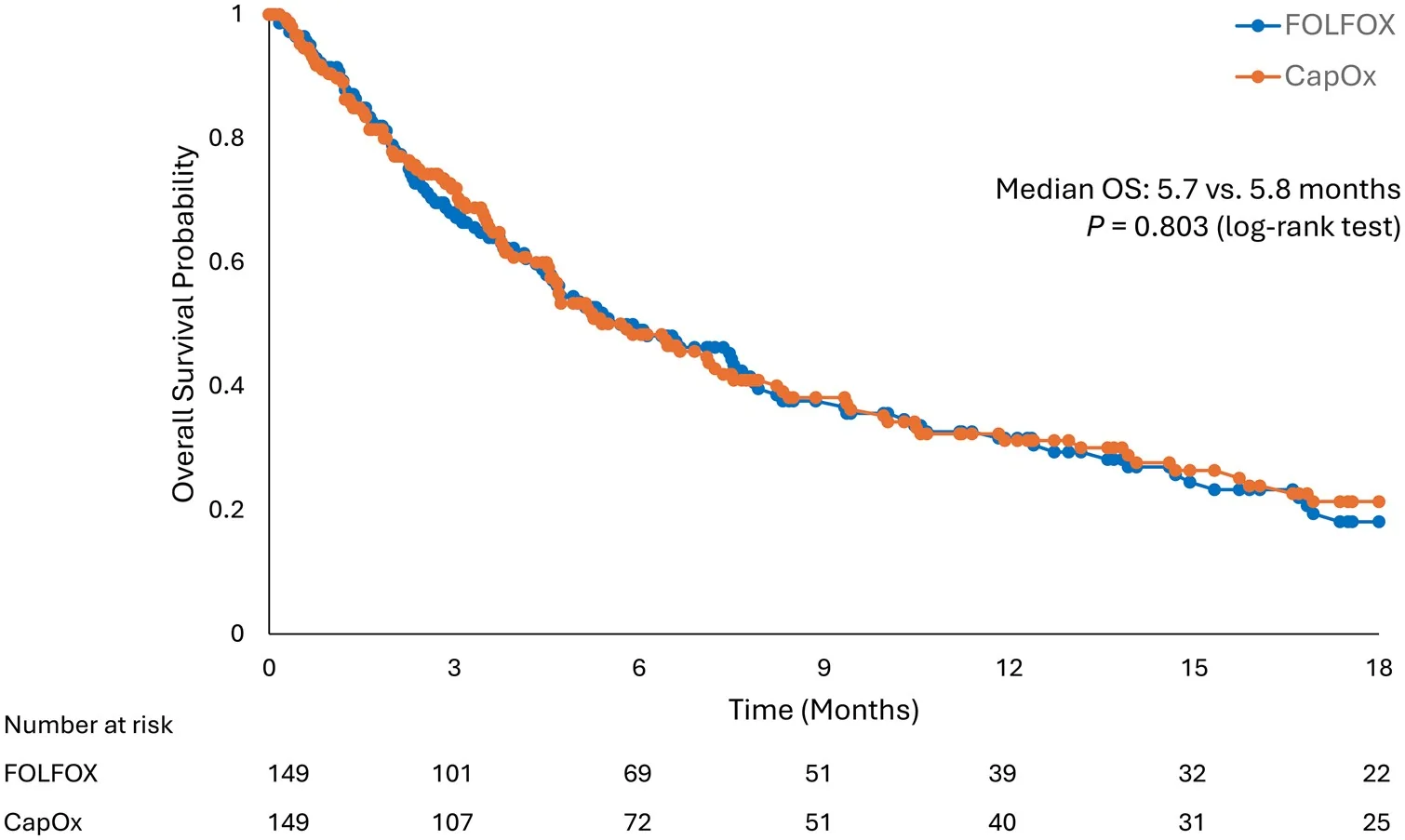
Overall survival of the propensity score-matched FOLFOX and CapOx cohorts. Kaplan-Meier curves comparing overall survival between patients treated with FOLFOX and CapOx after propensity score matching.

**Figure 5.**
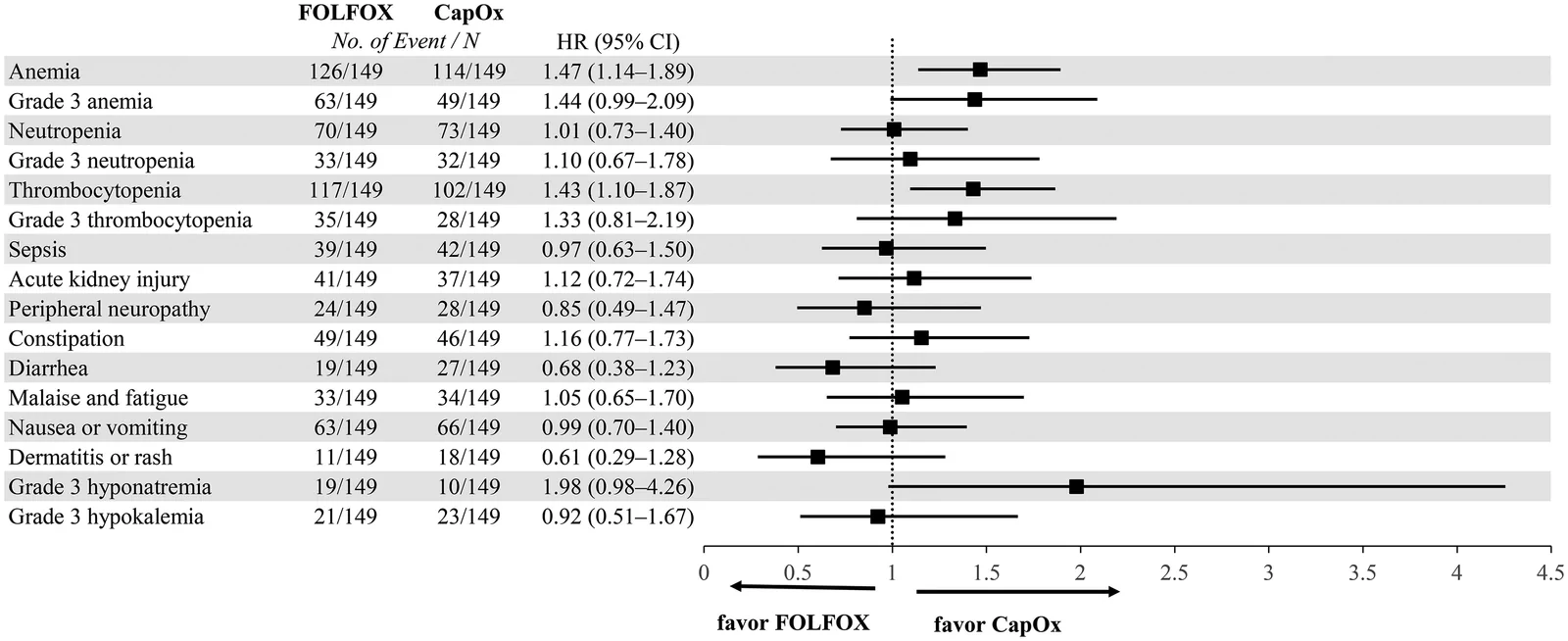
Adverse events in the propensity score-matched FOLFOX and CapOx cohorts. Forest plot comparing treatment-related adverse events between the FOLFOX and CapOx groups after propensity score matching.

## Discussion

This retrospective real-world study observed that FOLFIRI and CapOx achieved OS outcomes comparable to those achieved by FOLFOX in patients with advanced BTC who developed disease progression after first-line gemcitabine plus cisplatin. Following propensity score matching, the median OS was nearly identical between the FOLFOX and FOLFIRI groups (5.7 months vs 5.8 months) and between the FOLFOX and CapOx groups (5.7 months vs 5.8 months). Further, despite modest differences in specific toxicities among the regimens, the overall safety profiles were generally comparable.

In contemporary practice, second-line treatment selection for advanced BTC is increasingly guided by molecular profiling. Patients with actionable molecular alterations may be candidates for biomarker-directed therapies, whereas cytotoxic chemotherapy remains an important treatment option for patients without actionable alterations and for those unable to receive targeted therapy because of eligibility, access, tolerability, or other clinical considerations. Accordingly, the findings of the present study are most relevant to patients for whom cytotoxic chemotherapy remains an appropriate second-line treatment option.

Evidence supporting second-line treatment after progression on gemcitabine plus cisplatin in advanced BTC remains limited. To date, ABC-06 is the only phase III randomized trial in this setting, demonstrating a superior survival benefit with FOLFOX in comparison with active symptom control alone, with a median OS of 6.2 months vs 5.3 months.^19^ Based on these findings, FOLFOX has become the standard second-line chemotherapeutic regimen for advanced BTC. However, the control arm in ABC-06 received active symptom control rather than an alternative cytotoxic regimen. Thus, it remains unclear whether FOLFOX provides superior efficacy in comparison to other chemotherapy combinations. In addition, some patients develop persistent grade ≥2 peripheral neuropathy after first-line gemcitabine plus cisplatin and may not tolerate the additional neurotoxicity associated with oxaliplatin-containing therapy.^24^ Therefore, there remains an unmet clinical need for alternative second-line regimens for patients who maintain adequate performance status after the failure of first-line treatment.

Several studies have evaluated the efficacy of irinotecan-based regimens for advanced BTC. A randomized phase II trial by Choi et al.^20^ compared modified FOLFIRI (mFOLFIRI) and modified FOLFOX (mFOLFOX) in 118 patients who experienced disease progression after gemcitabine plus cisplatin. The median OS was 5.7 months in the mFOLFIRI arm and 6.3 months in the mFOLFOX arm, with comparable disease control rates of 64.0% and 66.7%, respectively. More recently, a multicenter Italian retrospective study involving 259 patients treated with FOLFOX (109 patients) or FOLFIRI (150 patients) also demonstrated similar outcomes between the 2 regimens, with median OS of 9.0 months and 9.9 months, respectively.^21^

Regarding safety, the present study found largely comparable overall adverse event profiles. However, FOLFOX was associated with a higher incidence of thrombocytopenia, whereas FOLFIRI was associated with a higher incidence of neutropenia. Similar patterns have been reported previously. In the phase II study by Choi et al., thrombocytopenia occurred more frequently with FOLFOX than with FOLFIRI (35.7% vs 15.5%, respectively). Similarly, studies on metastatic colorectal cancer have shown a higher incidence of neutropenia with FOLFIRI than with FOLFOX.^25^ These findings indicate distinct hematologic toxicity profiles for the 2 regimens, with thrombocytopenia being more prominent with FOLFOX and neutropenia occurring more frequently with FOLFIRI. Collectively, evidence from both prospective and real-world studies indicates that FOLFIRI and FOLFOX provide modest but comparable efficacy in patients with advanced BTC previously treated with gemcitabine plus cisplatin. Our results provide further real-world evidence to support the use of FOLFIRI as a reasonable alternative to FOLFOX in the second-line setting.

Liposomal irinotecan (nal-IRI), a nanoliposomal formulation designed to improve irinotecan pharmacokinetics and tumor delivery, has also emerged as a potential treatment option for advanced BTC. In the Korean phase IIb NIFTY trial, nal-IRI plus fluorouracil/leucovorin was associated with significantly longer progression-free survival (PFS) than fluorouracil/leucovorin alone (4.2 months vs 1.7 months).^26^ A subsequent meta-analysis of the ABC-06 and NIFTY trials demonstrated that fluoropyrimidine-based doublet chemotherapy was associated with a significantly reduced risk of death compared with control treatment in patients with advanced BTC.^27^ Although the German NALIRICC study reported less pronounced benefits, a pooled analysis of individual patient data of the 2 trials involving 278 patients demonstrated improved PFS with nal-IRI plus fluorouracil/leucovorin (3.6 months vs 1.8 months), with a trend toward improved OS (8.1 months vs 6.1 months).^26^^,^^28^

Nevertheless, evidence supporting nal-IRI in BTC remains limited to phase II studies, and no regulatory authority has formally approved nal-IRI for BTC. Consistent with real-world practice, the use of nal-IRI among patients with BTC was uncommon in the TriNetX network, precluding meaningful comparative analyses. Conventional irinotecan-based regimens are more commonly used in clinical practice. Therefore, the present study focused on conventional irinotecan-based therapy and provided additional real-world evidence to support the comparable efficacy of FOLFIRI, as observed in previous studies.

The current study also evaluated the efficacy of CapOx in patients with advanced BTC. Capecitabine is an oral fluoropyrimidine that has demonstrated comparable efficacy to infusional fluorouracil in several gastrointestinal malignancies while offering the convenience of oral administration.^29^ In the pivotal phase III NO16966 trial involving patients with metastatic colorectal cancer, CapOx demonstrated noninferior efficacy compared to FOLFOX4, with comparable OS.^23^ This established CapOx as a standard treatment option for metastatic colorectal cancer. These findings support the substitution of infusional fluorouracil with oral capecitabine in oxaliplatin-based combination regimens.

Similarly, in a pivotal phase III non-inferiority trial involving advanced BTC, CapOx demonstrated comparable efficacy to gemcitabine plus oxaliplatin (GEMOX), with a median PFS of 5.8 months vs 5.3 months and no significant difference in OS. Importantly, CapOx required significantly fewer hospital visits than GEMOX.^30^ However, evidence supporting the use of CapOx in the second-line setting remains limited. In a phase II study of 50 patients with advanced BTC who developed disease progression after gemcitabine plus cisplatin, CapOx achieved an objective response rate of only 14%; a disease control rate of 52%; and a median PFS and OS of 3.5 months and 7.6 months, respectively. However, the treatment was generally well tolerated, with thrombocytopenia and peripheral neuropathy being the most frequently reported adverse events.^31^

Since the establishment of FOLFOX as the standard second-line regimen based on the ABC-06 trial, replacing infusional fluorouracil with oral capecitabine has represented a clinically attractive strategy. FOLFOX requires a continuous 46-hour fluorouracil infusion, whereas CapOx allows oral administration of the fluoropyrimidine component and may therefore reduce the treatment burden associated with prolonged infusion. However, direct comparisons of CapOx and FOLFOX are lacking in this context. In the present real-world analysis, CapOx achieved survival outcomes nearly identical to those of FOLFOX, with generally comparable safety profiles. Thus, CapOx may represent a reasonable alternative for appropriately selected patients, particularly when treatment convenience and avoidance of prolonged fluorouracil infusion are important considerations.

From a clinical perspective, our findings suggest that the choice among these cytotoxic regimens may be guided less by expected differences in survival and more by individual patient characteristics, prior treatment-related toxicities, and practical considerations. FOLFIRI may be considered when further oxaliplatin exposure is undesirable, whereas CapOx may be an option for patients who prefer to avoid prolonged fluorouracil infusion. Conversely, the distinct hematologic toxicity profiles observed with FOLFOX and FOLFIRI may also inform regimen selection according to individual susceptibility to thrombocytopenia or neutropenia. These considerations support an individualized approach to second-line chemotherapy rather than a uniform preference for a single regimen.

The treatment landscape for advanced BTC has rapidly evolved with the emergence of molecularly targeted therapies.^32^ Several biomarker-directed therapies, including treatments targeting FGFR2 fusion, IDH1 mutation, HER2 amplification, BRAF V600E mutation, RET fusion, and NTRK fusion, have demonstrated clinically meaningful activity in molecularly selected populations.^16^^,^^18^^,^^33^^,^^34^ Recent molecular classifications have further highlighted the biological heterogeneity of cholangiocarcinoma and its therapeutic implications.^35^ Consequently, current international guidelines recommend comprehensive molecular profiling of patients with advanced BTC, whenever feasible.^17^ Nevertheless, actionable alterations and access to corresponding targeted therapies remain variable in clinical practice, underscoring the continued role of cytotoxic chemotherapy in appropriately selected patients.

Our case-identification strategy also differed from that of some prior registry-based BTC studies. Markussen et al. initially used a broad range of ICD-10 codes to maximize case capture and subsequently confirmed diagnoses through medical-record review.^36^ They found that restricting case identification to more BTC-specific ICD-10 codes captured nearly all confirmed BTC cases, supporting the use of more specific codes in registry-based analyses. In the present study, individual chart validation was not feasible within the TriNetX platform; therefore, we used a narrower set of ICD-10-CM codes (C22.1, C23, and C24.0) corresponding to intrahepatic cholangiocarcinoma, gallbladder cancer, and extrahepatic cholangiocarcinoma. Broader or non-BTC-specific codes, including those for ampullary cancer, were not included. This approach was intended to prioritize diagnostic specificity, although some residual misclassification cannot be excluded.

This study has some limitations. First, its retrospective observational design is inherently subject to selection bias and residual confounding despite the use of propensity score matching. Second, although propensity score matching was performed to balance the measured baseline characteristics, important clinical variables, including the Eastern Cooperative Oncology Group performance status and treatment dose intensity, were not consistently available in the TriNetX database. Ethnicity was not included in the prespecified study dataset and therefore could not be evaluated or incorporated into propensity score matching. Liver and bone metastases were not included among the metastatic-site covariates used for matching; in particular, distinguishing primary intrahepatic cholangiocarcinoma from secondary hepatic involvement using diagnosis codes alone may be challenging. Therefore, residual confounding related to the distribution and extent of metastatic disease cannot be excluded. In addition, subsequent lines of therapy after second-line treatment could not be reliably identified, precluding assessment of potential differences in third-line treatment between the matched cohorts and their potential influence on OS. Third, detailed TNM stage, radiologic response, PFS, and objective response rates could not be evaluated because these data were not routinely captured in the database. Additionally, comprehensive molecular profiling results were unavailable for a substantial proportion of patients, precluding subgroup analyses based on actionable genomic alterations. In addition, the study period spanned both the pre- and post-immunotherapy eras, during which first-line treatment for advanced BTC evolved substantially. Differences in first-line treatment patterns over time may therefore have introduced clinical heterogeneity and should be considered when interpreting our findings. Finally, the inherent limitations of the TriNetX platform, including missing data for selected variables and privacy restrictions on reporting small event counts, may have affected the precision of the analysis. Although follow-up duration was similar between the matched FOLFOX and CapOx cohorts, some difference was observed between the FOLFOX and FOLFIRI cohorts, and no additional adjustment for differential follow-up was performed. For threshold-defined laboratory covariates, patients without a recorded value were treated as not meeting the specified threshold, which may have resulted in residual misclassification of biomarker status. Therefore, our findings should be interpreted with caution and warrant validation. Prospective randomized controlled trials are warranted to validate the findings and to further define the optimal second-line chemotherapy strategy for advanced BTC.

## Conclusion

This real-world study shows that FOLFIRI and CapOx achieve OS outcomes comparable to those of FOLFOX, with modest differences in toxicity profiles, in patients with advanced BTC receiving second-line chemotherapy after progression on first-line gemcitabine plus cisplatin. Thus, FOLFIRI and CapOx may represent reasonable alternative doublet chemotherapy options in appropriately selected patients. Given the comparable survival outcomes observed among the 3 regimens, treatment selection may be guided primarily by individual patient characteristics, prior treatment-related toxicities, treatment convenience, and patient preference. Such an individualized approach may help optimize tolerability while maintaining clinical benefits.

## Supplementary Material

oyag424_Supplementary_Data

## Data Availability

The data used in this study were collected on February 17, 2026, from the TriNetX Global Collaborative Network, which provided access to electronic medical records (diagnoses, procedures, medications, laboratory values) from approximately 179 537 628 patients across 172 healthcare organizations. Due to TriNetX data-use and licensing restrictions, individual-level datasets are not publicly available, but aggregated results and analytic specifications are available from the corresponding author on reasonable request.
