# Supplementary material for: Real-world comparative effectiveness of FOLFOX, FOLFIRI, and CapOx as second-line chemotherapy for advanced biliary tract cancer
Source: Oncologist. 2026 Oct 21;31(11):oyag424. doi: 10.1093/oncolo/oyag424 (PMC13730269; doi:10.1093/oncolo/oyag424)
Supplement: Supplementary file 1 [file oyag424_supplementary_data.docx]

**Supplementary file**

**Table S1.** Details of the diagnostic and procedural query codes used in the analysis

| **Diagnosis** | | |
| --- | --- | --- |
| Biliary tract cancer | ICD-10-CM: C23 | Malignant neoplasm of gallbladder |
|  | ICD-10-CM: C22.1 | Intrahepatic bile duct carcinoma |
|  | ICD-10-CM: C24.0 | Malignant neoplasm of extrahepatic bile duct |
| Sites of metastasis | ICD-10-CM: C77 | Secondary and unspecified malignant neoplasm of lymph nodes |
|  | ICD-10-CM: C78 | Secondary malignant neoplasm of respiratory and digestive organs |
|  | ICD-10-CM: C79 | Secondary malignant neoplasm of other and unspecified sites |
| Diabetes mellitus | ICD-10-CM: E08-E13 |  |
| Ischemic heart diseases | ICD-10-CM: I20-I25 |  |
| Chronic lower respiratory diseases | ICD-10-CM: J40-J4A |  |
| Chronic kidney disease | ICD-10-CM: N18 |  |
| **Medications** | | |
| Gemcitabine | RxNorm: 12574 | Gemcitabine |
|  | HCPCS: J9201, J9196, J9199, J9198 | Gemcitabine |
| Cisplatin | RxNorm: 2555 | Cisplatin |
|  | HCPCS: J9060 | Cisplatin |
| Oxaliplatin | RxNorm: 32592 | Oxaliplatin |
|  | HCPCS: J9263 | Oxaliplatin |
| Fluorouracil | RxNorm: 4492 | Fluorouracil |
|  | HCPCS: J9190 | Fluorouracil |
| Irinotecan | RxNorm: 51499 | Irinotecan |
|  | HCPCS: J9206 | Irinotecan |
| Capecitabine | RxNorm: 194000 | Capecitabine |
|  | HCPCS: J8520, J8521 | Capecitabine |
| Ivosidenib | RxNorm: 2049873 | Ivosidenib |
| Pemigatinib | RxNorm: 2359268 | Pemigatinib |
| Dabrafenib | RxNorm: 1424911 | Dabrafenib |
| Trametinib | RxNorm: 1425099 | Trametinib |
| Trastuzumab | RxNorm: 224905 | Trastuzumab |
| Trastuzumab deruxtecan | RxNorm: OMOP5051437 | Trastuzumab deruxtecan |
| Pertuzumab | RxNorm: 1298944 | Pertuzumab |
| Selpercatinib | RxNorm: 2370147 | Selpercatinib |
| Larotrectinib | RxNorm: 2105628 | Larotrectinib |
| Entrectinib | RxNorm: 2197862 | Entrectinib |
| Olaparib | RxNorm: 1597582 | Olaparib |
| Niraparib | RxNorm: 1918231 | Niraparib |
| Rucaparib | RxNorm: 1862579 | Rucaparib |
| Pembrolizumab | RxNorm: 1547545 | Pembrolizumab |
| **Outcomes (Diagnosis & Lab exams with LOINC)** | | |
| Deceased |  |  |
| Anemia  Grade 3 anemia | LOINC: 718-7  TNX: 9014 | Anemia: haemoglobin ≤ 12.0 g/dL  Grade 3 anemia: haemoglobin ≤ 8.0 g/dL |
| Neutropenia  Grade 3 neutropenia | LOINC: 26499-4  TNX: 9018 | Neutropenia ≤ 2,500/mm^3^  Grade 3 neutropenia: neutrophil ≤ 1,000/mm^3^ |
| Thrombocytopenia  Grade 3 thrombocytopenia | LOINC: 26515-7  TNX: 9020 | Thrombocytopenia: platelet ≤ 150,000/mm^3^  Grade 3 thrombocytopenia: platelet ≤ 50,000/mm^3^ |
| Sepsis | ICD-10-CM: A40  ICD-10-CM: A41  ICD-10-CM: N39.0  ICD-10-CM: R65.2 | Suspected infection with ≥ 2 of 3 clinical variables  -Glasgow Coma Scale score of ≤ 13  -Systolic blood pressure of ≤ 100 mm Hg  -Respiratory rate ≥ 22/min |
| Acute kidney injury | ICD-10-CM: N17  ICD-10-CM: N28 | Increase in serum creatinine by ≥ 0.3 mg/dL (≥26.5 μmol/L) within 48 h or increase in serum creatinine to ≥ 1.5 times the baseline of the prior 7 days or urine volume < 0.5 mL/kg/h for at least 6 h. |
| Peripheral neuropathy | ICD-10-CM: G62.0  ICD-10-CM: G62.9  ICD-10-CM: G63 | Drug-induced polyneuropathy  Polyneuropathy, unspecified  Polyneuropathy in diseases classified elsewhere |
| Constipation | ICD-10-CM: K59.0 |  |
| Diarrhea | ICD-10-CM: R19.7  ICD-10-CM: K52.89 |  |
| Malaise and Fatigue | ICD-10-CM: R53.83 |  |
| Nausea and vomiting | ICD-10-CM: R11 |  |
| Dermatitis/Rash | ICD-10-CM: L27.0  ICD-10-CM: L27.1  ICD-10-CM: L30.0  ICD-10-CM: L30.8  ICD-10-CM: L30.9  ICD-10-CM: L53.9  ICD-10-CM: R21 |  |
| Hyponatremia | LOINC: 2951-2  TNX: 9029 | Grade 3 hyponatremia: sodium ≤ 125.00 mmol/L |
| Hypokalemia | LOINC: 2823-3  TNX: 9028 | Grade 3 hypokalemia: potassium ≤ 2.90 mmol/L |

The codes used to identify patient diagnosis, medications, and laboratory results were based on the International Classification of Disease 10^th^ Clinical Modification (ICD-10-CM), Logical Observation Identifier Name and Codes (LOINC), RxNorm, and Healthcare Common Procedure Coding System (HCPCS).

**Fig. S1** Distribution of propensity score matching. Distribution (A) before and (B) after propensity score matching for the FOLFOX and FOLFIRI groups. FOLFOX= fluorouracil, leucovorin, and oxaliplatin; FOLFIRI= fluorouracil, leucovorin, and irinotecan.

Alt-text: Distribution plots of propensity scores for the FOLFOX and FOLFIRI cohorts before and after propensity score matching.

**Fig. S2** Distribution of propensity score matching. Distribution (A) before and (B) after propensity score matching for the FOLFOX and CapOx groups. FOLFOX= fluorouracil, leucovorin, and oxaliplatin; CapOx= capecitabine and oxaliplatin.

Alt-text: Distribution plots of propensity scores for the FOLFOX and CapOx cohorts before and after propensity score matching.
